# Dexmedetomidine alleviates pulmonary fibrosis through the ADORA2B-Mediated MAPK signaling pathway

**DOI:** 10.1186/s12931-023-02513-3

**Published:** 2023-08-29

**Authors:** Xiaofan Lai, Yingying Lin, Shaojie Huang, Lvya Pu, Qihao Zeng, Zhongxing Wang, Wenqi Huang

**Affiliations:** 1 Department of Anesthesiology, The First Affiliated Hospital, Sun Yat-sen University, Guangzhou, China; 2 Zhongshan School of Medicine, Sun Yat-sen University, Guangzhou, China

**Keywords:** Idiopathic pulmonary fibrosis, Dexmedetomidine, Myofibroblasts, ADORA2B

## Abstract

**Background:**

Idiopathic pulmonary fibrosis (IPF) is a chronically progressive fibrotic pulmonary disease characterized by an uncertain etiology, a poor prognosis, and a paucity of efficacious treatment options. Dexmedetomidine (Dex), an anesthetic-sparing alpha-2 adrenoceptor (α2AR) agonist, plays a crucial role in organ injury and fibrosis. However, the underlying mechanisms of IPF remain unknown.

**Methods:**

In our study, the role of Dex in murine pulmonary fibrosis models was determined by Dex injection intraperitoneally in vivo. Fibroblast activation and myofibroblast differentiation were assessed after Dex treatment in vitro. The activation of MAPK pathway and the expression of Adenosine A2B receptor (ADORA2B) were examined in lung myofibroblasts. Moreover, the role of ADORA2B in Dex suppressing myofibroblast differentiation and pulmonary fibrosis was determined using the ADORA2B agonist BAY60-6583.

**Results:**

The results revealed that Dex could inhibit Bleo-induced pulmonary fibrosis in mice. In vitro studies revealed that Dex suppressed TGF-β-mediated MAPK pathway activation and myofibroblast differentiation. Furthermore, Dex inhibits myofibroblast differentiation and pulmonary fibrosis via downregulating ADORA2B expression.

**Conclusions:**

Our findings suggest Dex as a potential therapeutic agent for pulmonary fibrosis. Dex may alleviate lung fibrosis and myofibroblast differentiation through the ADORA2B-mediated MAPK signaling pathway.

**Supplementary Information:**

The online version contains supplementary material available at 10.1186/s12931-023-02513-3.

## Introduction

Idiopathic pulmonary fibrosis (IPF) is the most prevailing interstitial lung disease with an unfavorable prognosis [1]. It is primarily distinguished by continuous epithelial cell death, myofibroblast activation and excessive accumulation of extracellular collagen in the lung interstitium [2, 3]. Though the drugs nintedanib [4] and pirfenidone [5] were approved by FDA for IPF treatment, both drugs show apparent toxicities. Therefore, it is in urgent need to identify the mechanisms underlying IPF progression and develop new approaches.

Dexmedetomidine (Dex), an anesthetic-sparing alpha-2 adrenoceptor (α2AR) agonist, exhibits anesthetic-sparing, is widely used for sedation and anesthesia clinically [6, 7]. Abundant studies showed that Dex exerts protective effects on injury of several organs including liver [8], heart [9, 10], lung [11, 12], kidney [13, 14], intestine [15] and so on. Honggang Fan and colleagues found that Dex markedly induced MKP-1 activation and inhibited NF-κB pathway, thus effectively attenuating acute stress-induced liver injury in rats [8]. Hong Liu group reported that perioperative use of Dexmedetomidine could reduce the occurrence of complications and mortality in patients those undergo cardiac surgery [10]. Besides, Dex alleviates intestinal ischemia/reperfusion-induced lung injury mainly through the increase of cannabinoid receptor 2 and the regulation of PI3K/Akt signaling pathway [12]. It was proved that a single dose of Dex exerted protective effects on acute kidney injury and chronic kidney disease via suppressing the senescence of tubular cells, providing a new potential therapy for renal injury patients [14]. Moreover, Dex improves intestinal epithelial barrier disruption in endotoxemic rats mainly through ameliorating mucosal cell death and tight junctional damage [15]. However, whether Dex affects the development of IPF and its underlying mechanisms remain unclear.

Adenosine A2B receptor (ADORA2B), one of the four subtypes of adenosine receptors, is a member of the G protein-coupled receptor superfamily [16]. ADORA2B was widely reported to play a critical role in ischaemia-reperfusion injury, cancer growth, immune and inflammatory response. and fibrosis of a variety of organs [17]. Emerging evidence supports ADORA2B as the major regulator of the trajectory of pulmonary fibrosis [18]. Michael R Blackburn and colleagues showed that ADORA2B antagonism could significantly downregulate proinflammatory cytokines and chemokines and alleviate lung fibrosis, suggesting that ADORA2B signaling plays a central part in pulmonary injury and fibrosis in vivo [19]. Zhigang Zhang group reported that Multi-walled carbon nanotube (MWCNT)-induced lung fibrosis facilitated lung myofibroblasts differentiation in an ADORA2B-dependent manner, and the ADORA2B antagonist CVT-6883 significantly reduced the lung fibrosis degree in MWCNT-treated mice [20]. In addition, ADORA2B was able to regulate the cAMP/PKA and MAPK pathways in human lung epithelial cells, shifting the balance between Epithelial-Mesenchymal Transition (EMT) activation or inhibition, which highlights the role of ADORA2B in the modulation of EMT process [21]. However, whether Dex affect pulmonary fibrosis largely via ADORA2B remains unknown.

In the present study, we investigated the effects of Dex on pulmonary fibrosis through pulmonary fibrosis murine models. We explored whether Dex is involved in regulating the activation of lung fibroblasts. In addition, we also studied the potential role of Dex in modulating MAPK signaling pathway and ADORA2B expression.

## Results

### Dex attenuates pulmonary fibrosis in experimental mouse models

**Fig. 1:**
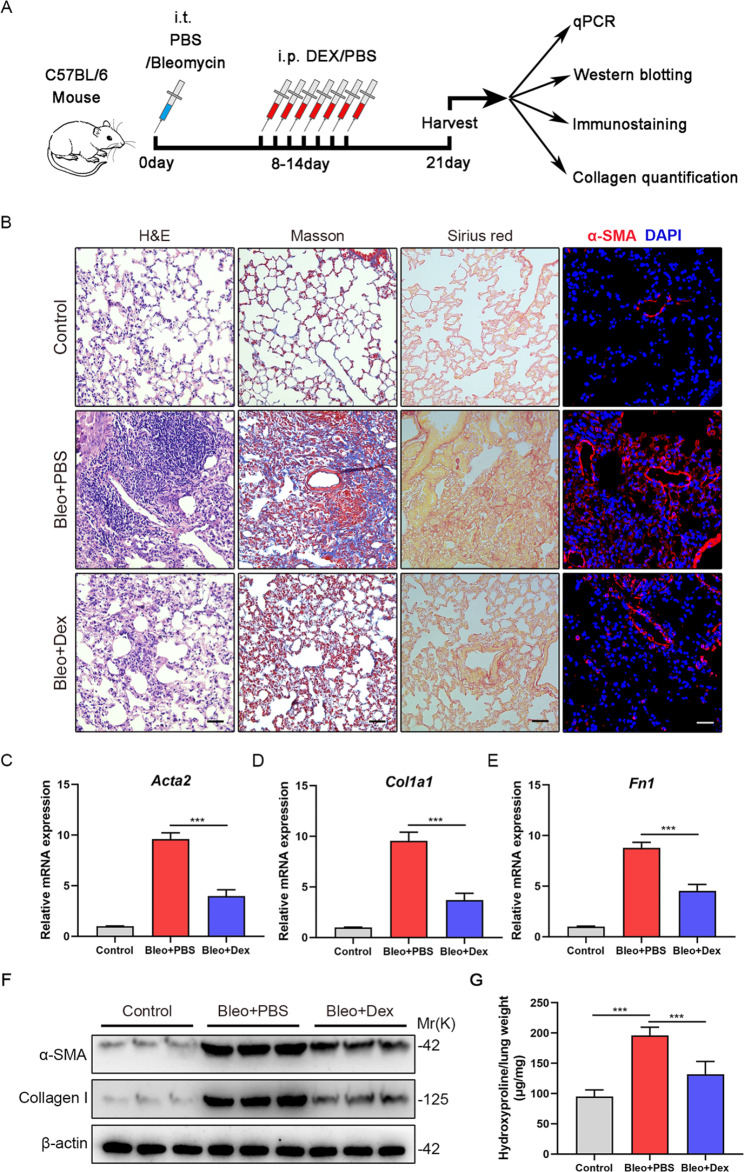
Dex attenuates pulmonary fibrosis in experimental mice models. (**A**) Experimental design. 8-week-old C57BL/6 mice were injected with bleomycin (3U/kg) or PBS intratracheally. Eight days later, the mice were intraperitoneal injected with Dex or PBS of equal volume. Lung tissues were collected for experiments 21 days after bleomycin administration. (**B**) Representative H&E staining, Masson’s trichrome staining, Sirius red staining and immunofluorescence images of lung sections using anti-α-SMA (red) antibodies from the different groups (n = 5 mice per group; five fields assessed per sample). Scale bars: 50 μm. (**C**) qPCR analysis of *Acta2* mRNA expression in the lungs of C57/BL6 mice from the different groups (n = 6 mice per group). (**D**) qPCR analysis of *Col1a1* mRNA expression in the lungs of C57/BL6 mice from the different groups (n = 6 mice per group). (**E**) qPCR analysis of *Fn1* mRNA expression in the lungs of C57/BL6 mice from the different groups (n = 6 mice per group). (**F**) Western blot analysis of α-SMA and Collagen I expression level in lungs of C57/BL6 mice from the different groups. (**G**) Hydroxyproline levels in lungs of C57/BL6 mice from the different groups (n = 6 mice per group). Data are presented as the mean ± SD of three independent experiments; ***P < 0.001; one-way ANOVA and Tukey’s multiple comparisons test

In order to investigate the role of Dex in the pathogenesis of lung fibrosis, we conducted a Bleomycin (Bleo)-induced lung fibrosis mice model and from day 8th mice were intraperitoneally injected Dex or PBS of equal volume continuously for 7 days. The workflow of our animal experiments was shown in Fig. 1A. At 21 days after Bleo administration, the mice lung tissues were harvested for experiments and analysis. The results showed that the inflammation and fibrosis severity in lungs from Bleo + Dex group was significantly less than that from Bleo + PBS group, as evidenced by H&E, sirius red and Masson Trichrome staining (Fig. 1B). Immunofluorescence staining revealed that there was less α-SMA positive area in lungs from Bleo + Dex group relative to control (Fig. 1B). The mRNA expression of Acta2, Col1a1 and Fn1 increased significantly in lungs from Bleo + PBS group, while these were relatively reduced after Dex injection (Fig. 1C-E). Consistently, the protein levels of α-SMA and collagen I in lung tissues from the Bleo + Dex group were markedly lower than those in lung tissues from the Bleo + PBS group (Fig. 1F). In addition, the administration of Dex could strongly alleviate bleomycin-induced pulmonary fibrosis as evidenced by hydroxyproline assays (Fig. 1G). Taken together, Dex exerts protective effects on Bleo-induced pulmonary fibrosis in mice.

### Dex inhibits lung myofibroblast differentiation induced by TGF-β

**Fig. 2:**
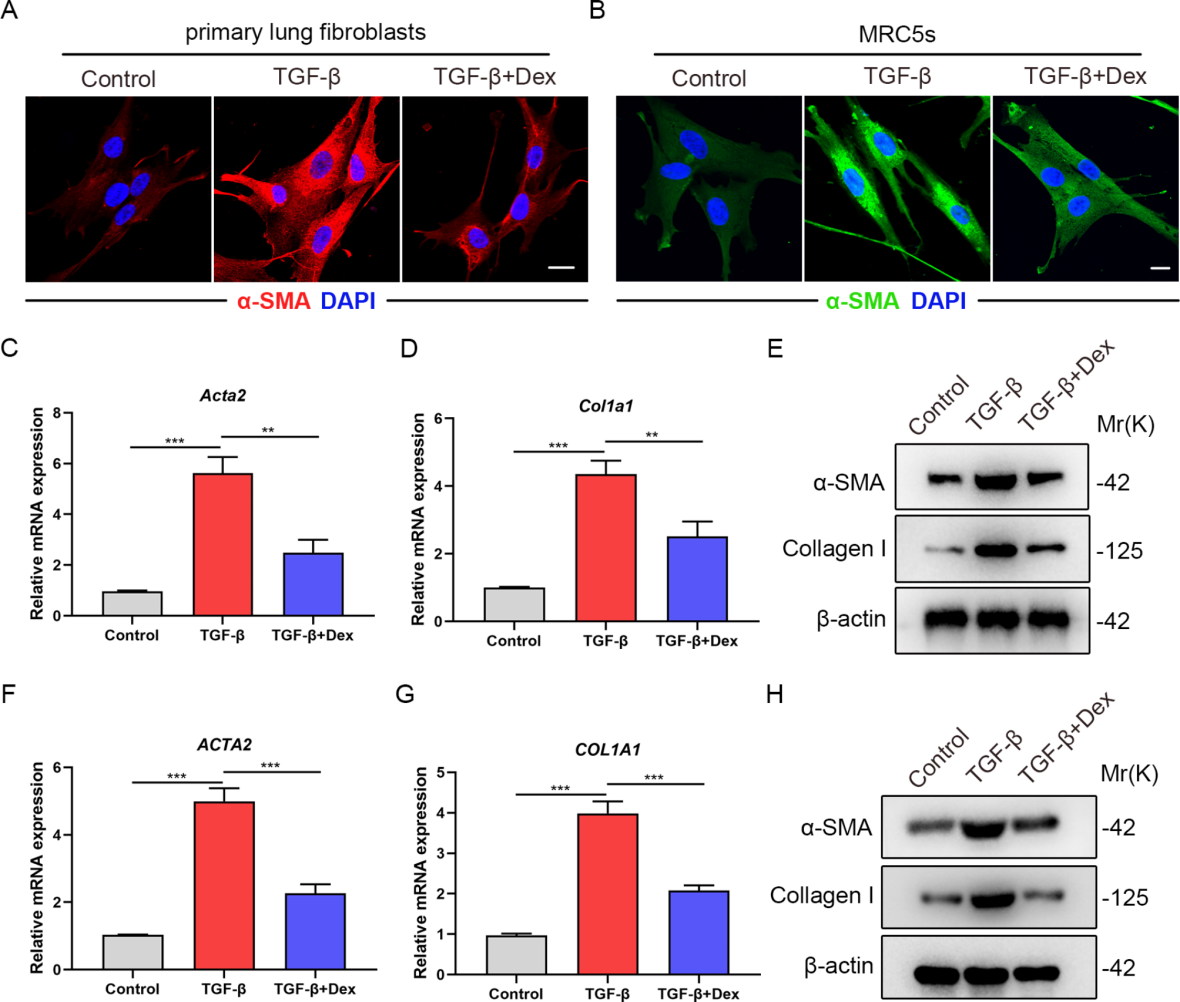
Dex inhibits lung myofibroblast differentiation induced by TGF-β. (**A**) Representative immunofluorescence images of primary mouse lung fibroblasts and treated with or without Dex using anti-α-SMA (red) antibodies. Scale bar: 20 μm. (**B**) Representative immunofluorescence images of MRC5s and treated with or without Dex using anti-α-SMA (green) antibodies. Scale bar: 20 μm. (**C**) qPCR analysis of *Acta2* mRNA levels in primary mouse lung fibroblasts subjected to Dex treatment with or without TGF-β (5 ng/ml) (n = 3 per group). (**D**) qPCR analysis of *Col1a1* mRNA levels in primary mouse lung fibroblasts subjected to Dex treatment with or without TGF-β (5 ng/ml) (n = 3 per group). (**E**) Western blot analysis of α-SMA and Collagen I protein expression in primary mouse lung fibroblasts subjected to Dex treatment with or without TGF-β (5 ng/ml). (**F**) qPCR analysis of *ACTA2* mRNA levels in MRC5s subjected to Dex treatment with or without TGF-β (5 ng/ml) (n = 3 per group). (**G**) qPCR analysis of *COL1A1* mRNA levels in MRC5s subjected to Dex treatment with or without TGF-β (5 ng/ml) (n = 3 per group). (**H**) Western blot analysis of α-SMA and Collagen I protein expression in MRC5s subjected to Dex treatment with or without TGF-β (5 ng/ml). Data are presented as the mean ± SD of three independent experiments; **P < 0.01, ***P < 0.001; one-way ANOVA and Tukey’s multiple comparisons test

It was widely acknowledged that myofibroblasts perform an important constructive role in the progression of pulmonary fibrosis [22]. Next we explored whether Dex treatment influence the activation of primary lung fibroblasts from mice and human fetal lung fibroblasts cell line-MRC5s in vitro. Through immunofluorescence staining we observed that α-SMA was significantly increased with TGF-β stimulation, while it was relatively reduced after Dex treatment within cells (Fig. 2A-B). RT-qPCR and western blot analysis revealed that Dex could inhibit the TGF-β-induced upregulation of α-SMA and Collagen I expression in primary lung fibroblasts (Fig. 2C-E). Consistently, Dex could also inhibit the increase of α-SMA and Collagen I expression induced by TGF-β in MRC5s, suggesting that Dex played an inhibitory role in the TGF-β-mediated myofibroblast differentiation (Fig. 2F-H). Overall, Dex alleviates lung myofibroblast differentiation induced by TGF-β in vitro.

### Dex suppressed the activation of MAPK signaling pathway induced by TGF-β

**Fig. 3:**
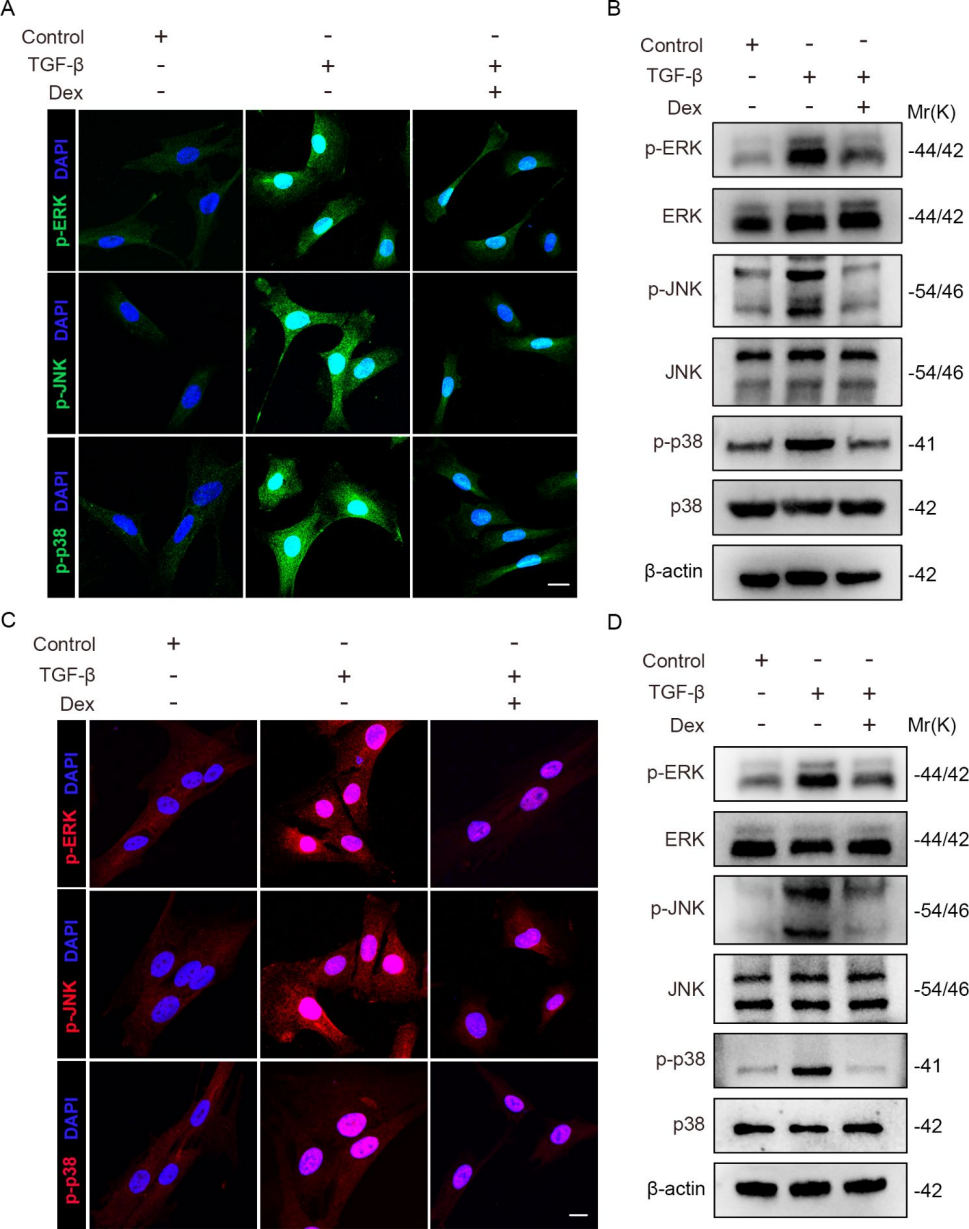
Dex inhibits the activation of MAPK signaling pathway induced by TGF-β. (**A**) Representative immunofluorescence images of primary mouse lung fibroblasts subjected to Dex treatment using anti-p-ERK, anti-p-JNK or anti-p-p38 (green) antibodies with or without TGF-β (5 ng/ml). Scale bar: 20 μm. (**B**) Western blot analysis of p-ERK, ERK, p-JNK, JNK, p-p38 and p38 expression level in primary mouse lung fibroblasts subjected to Dex treatment with or without TGF-β (5 ng/ml). (**C**) Representative immunofluorescence images of MRC5s subjected to Dex treatment using anti-p-ERK, anti-p-JNK or anti-p-p38 (red) antibodies with or without TGF-β (5 ng/ml). Scale bar: 20 μm. (**D**) Western blot analysis of p-ERK, ERK, p-JNK, JNK, p-p38 and p38 expression level in MRC5s subjected to Dex treatment with or without TGF-β (5 ng/ml). Data are presented as the mean ± SD of three independent experiments; one-way ANOVA and Tukey’s multiple comparisons test

Mitogen-activated protein kinases (MAPK), a member of the serine-threonine kinases superfamily, are involved in specific biologic responses and the pathogenesis [23] of different diseases. Now there are three recognized MAPK subfamilies including extracellular signal-regulated kinase (ERK), p38 MAPK, and c-Jun-N-terminal kinase (JNK) [24]. Accumulating evidence shows that MAPK signaling pathway plays a critical role in lung fibroblast activation and extracellular matrix accumulation [25, 26]. To determine whether Dex inhibits the lung myofibroblast differentiation is related with the MAPK signaling pathway, we first assessed the activation of MAPK signaling pathway in mouse primary lung fibroblasts with Dex treatment. In vitro experiments revealed that compared with the control group, TGF-β induced the phosphorylation of p-ERK, p-JNK and p-p38 as well as their nuclear translocation, and Dex showed an inhibitory effect on MAPK phosphorylation and nuclear translocation (Fig. 3A). Western blot analysis proved that the phosphorylation of p-ERK, p-JNK and p-p38 in Dex-treated cells were markedly decreased compared with TGF-β group cells (Fig. 3B). To further confirm the results, we also used MRC5s for examination. Consistently, we found that MAPK phosphorylation and nuclear translocation was apparently reduced after Dex treatment relative to control (Fig. 3C). p-ERK, p-JNK and p-p38 phosphorylation in Dex-treated group were also lower than only TGF-β group (Fig. 3D). In addition, we also conducted a Bleomycin-induced lung fibrosis mice model and from day 8th mice were intraperitoneally injected Dex or PBS of equal volume continuously for 7 days as shown in Fig. 1A. Immunofluorescence staining revealed that compared with the Control group, Bleomycin strongly induced the phosphorylation of p-ERK, p-JNK and p-p38 in lungs. While MAPK phosphorylation of lungs in the Bleo + Dex group was strongly inhibited compared to the Bleo + PBS group (Supplementary Fig. 1). These results are consistent with the in vitro experiments. Altogether, the above data suggested that Dex suppress the activation of MAPK signaling pathway induced by TGF-β in lung myofibroblasts.

### Dex downregulates the expression of ADORA2B in lung myofibroblasts

**Fig. 4:**
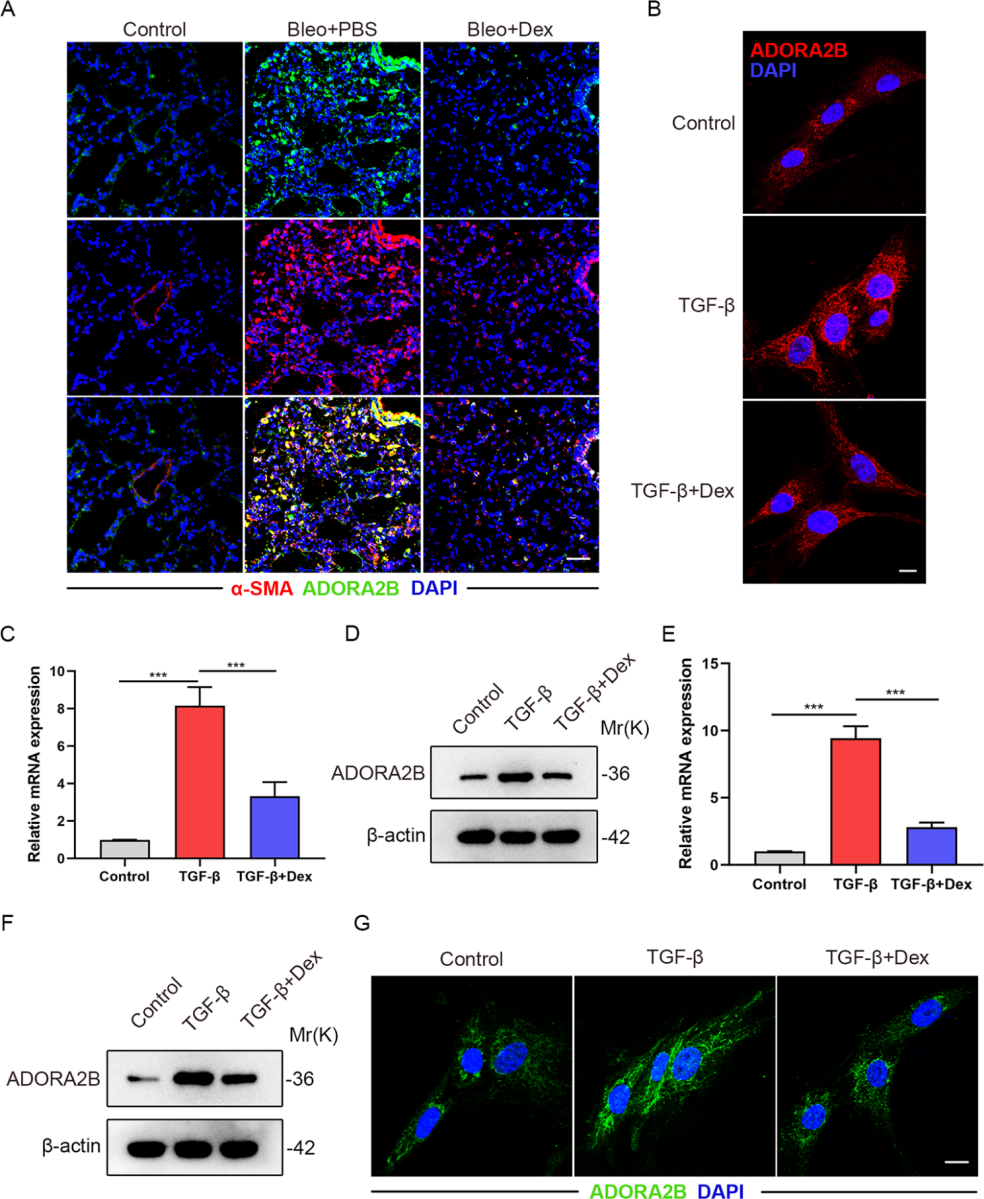
Dex downregulates the expression of ADORA2B in lung myofibroblasts.(**A**) Representative immunofluorescence images of lung sections using anti-α-SMA (red) and anti-ADORA2B (green) antibodies from the different groups. Scale bars: 50 μm. (**B**) Representative immunofluorescence images of primary mouse lung fibroblasts subjected to Dex treatment with or without TGF-β (5 ng/ml) using anti-ADORA2B (red) antibody. Scale bars: 10 μm. (**C**) qPCR analysis of *Adora2b* mRNA levels in primary mouse lung fibroblasts subjected to Dex treatment with or without TGF-β (5 ng/ml) (n = 3 per group). (**D**) Western blot analysis of ADORA2B expression level in primary mouse lung fibroblasts subjected to Dex treatment with or without TGF-β (5 ng/ml). (**E**) qPCR analysis of *ADORA2B* mRNA levels in MRC5s subjected to Dex treatment with or without TGF-β (5 ng/ml) (n = 3 per group). (**F**) Western blot analysis of ADORA2B expression level in MRC5s subjected to Dex treatment with or without TGF-β (5 ng/ml). (**G**) Representative immunofluorescence images of MRC5s subjected to Dex treatment with or without TGF-β (5 ng/ml) using anti-ADORA2B (green) antibody from the different groups. Scale bars: 20 μm. Data are presented as the mean ± SD of three independent experiments; ***P < 0.001; unpaired t test and one-way ANOVA and Tukey’s multiple comparisons test

It was reported that the adenosine receptor ADORA2B in different cell types was able to activate MAPK pathways including ERK, JNK and p38 [17, 27]. ADORA2B was known to be the major regulator of the formation of lung myofibroblasts and the development of lung fibrosis [18]. To determine if Dex exerts inhibitory effects on the activation of lung fibroblasts through the regulation of ADORA2B, we first adopted immunofluorescence staining and observed that ADORA2B expression was markedly decreased in lungs from Bleo + Dex group compared with those from Bleo + PBS group (Fig. 4A). Next, we isolated mouse primary lung fibroblasts and detected that the expression of ADORA2B was relatively decreased in TGF-β + Dex group compared with those in TGF-β group by immunofluorescence staining (Fig. 4B). Besides, the ADORA2B mRNA and protein expression levels were reduced after Dex stimulation relative to control (Fig. 4C-D). Moreover, to study the role of ADORA2B in Dex regulating myofibroblast differentiation, we applied MRC5s for analysis and confirmed that ADORA2B mRNA and protein expression were upregulated after TGF-β stimulation, but they are relatively decreased when treated with Dex (Fig. 4E-G). The above experimental results indicated that Dex downregulates the expression of ADORA2B in lung myofibroblasts.

### Dex inhibits TGF-β-mediated MAPK pathway activation and myofibroblast differentiation via regulating ADORA2B

**Fig. 5:**
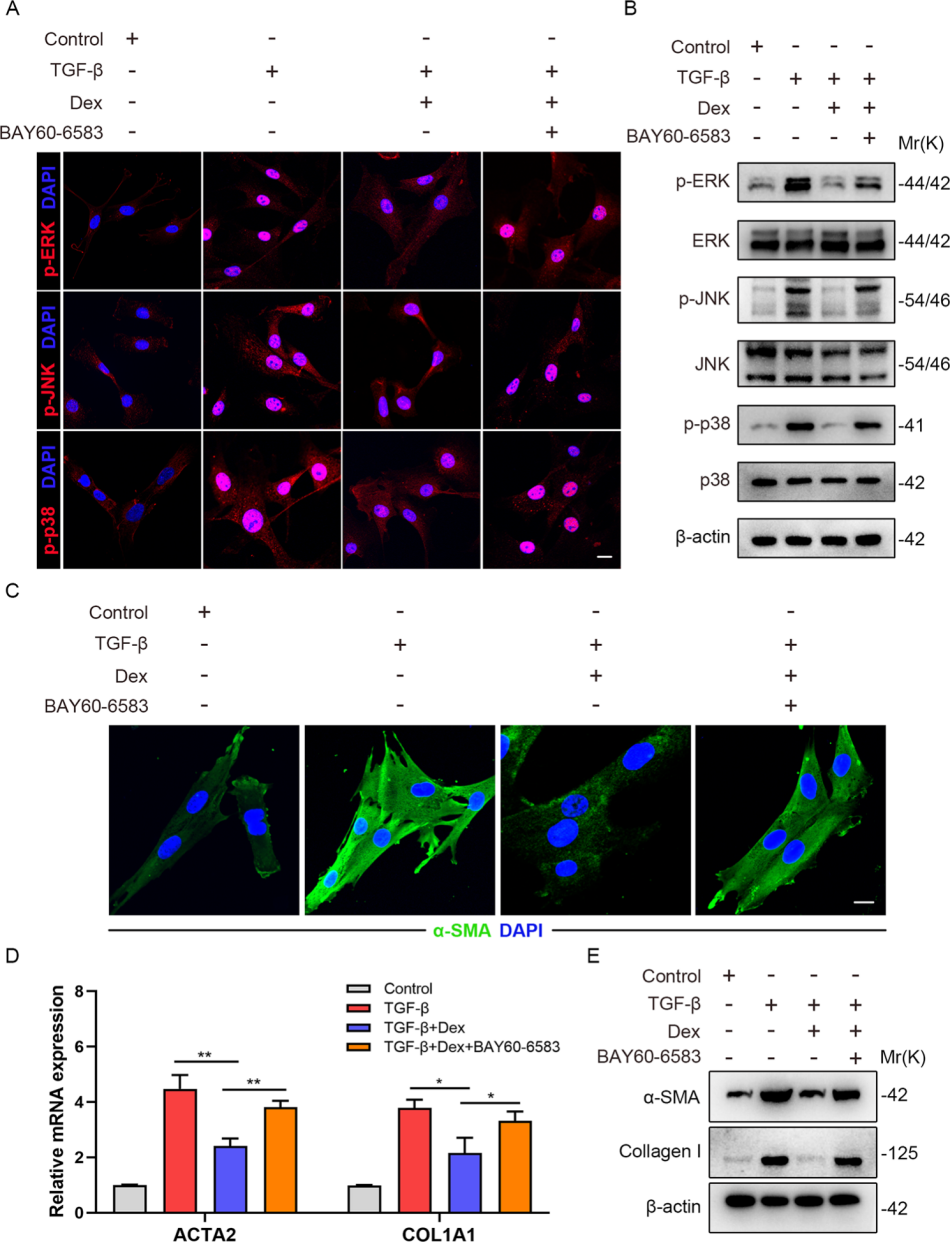
Dex inhibits TGF-β-mediated MAPK pathway activation and myofibroblast differentiation via regulating ADORA2B. (**A**) Representative immunofluorescence images of MRC5s subjected to Dex and BAY60-6583 treatment with or without TGF-β (5 ng/ml) using anti-p-ERK, anti-p-JNK or anti-p-p38 (red) antibodies. Scale bar: 20 μm. (**B**) Western blot analysis of p-ERK, ERK, p-JNK, JNK, p-p38 and p38 expression level in MRC5s subjected to Dex and BAY60-6583 treatment with or without TGF-β (5 ng/ml). (**C**) Representative immunofluorescence images of MRC5s subjected to Dex and BAY60-6583 treatment with or without TGF-β (5 ng/ml) using anti-α-SMA (green) antibodies. Scale bar: 20 μm. (**D**) qPCR analysis of *ACTA2* and *COL1A1* mRNA expression MRC5s subjected to Dex and BAY60-6583 treatment with or without TGF-β (5 ng/ml). (**E**) Western blotting of α-SMA and Collagen I expression levels in MRC5s subjected to Dex and BAY60-6583 treatment with or without TGF-β (5 ng/ml). Data are presented as the mean ± SD of three independent experiments; *P < 0.05, **P < 0.01; one-way ANOVA and Tukey’s multiple comparisons test

To determine if Dex could inhibit the activation of lung fibroblasts through regulating ADORA2B, we next adopted the highly selective ADORA2B agonist BAY60-6583 simultaneously for rescue experiments in vitro. The results showed that the relatively reduction of ERK, JNK and p38 phosphorylation as well as their nuclear translocation after Dex treatment with TGF-β stimulation could be largely restored by adding BAY60-6583 in MRC5s (Fig. 5A). Consistently, via western blot analysis we proved that Dex could downregulate the phosphorylation of p-ERK, p-JNK and p-p38 in TGF-β-treated cells, which was strongly blocked by BAY60-6583 treatment in MRC5s and primary lung fibroblasts (Fig. 5B and supplementary Fig. 2). The above data suggested that Dex inhibits TGF-β-mediated MAPK pathway activation possibly by regulating ADORA2B. Similarly, Dex suppressed the TGF-β-induced increase of α-SMA, which could be strongly rescued by BAY60-6583 (Fig. 5C). Furthermore, Dex-mediated inhibition on the transcript and protein levels of α-SMA and Collagen I was also apparently restored by BAY60-6583 treatment (Fig. 5D-E). Through these above experiments, we confirmed Dex suppressed TGF-β-mediated MAPK pathway activation and myofibroblast differentiation via modulating ADORA2B.

### The role of Dex in TGF-β-induced differentiation of lung myofibroblasts is dependent on α2A adrenoreceptor

Three subtypes of α2 adrenoreceptor (α2 AR) were identified so far including α2A AR, α2B AR and α2C AR [28]. Abundant evidence suggests that α2A AR plays a vital role in the regulation of the analgesic, sedative and hypotensive actions of Dex [29–31]. To further investigate whether the effect of Dex on TGF-β-induced differentiation of lung myofibroblasts is dependent on α2A AR, we used BRL44408, Imiloxan HCl and JP-1302, which is the selective α2A AR, α2B AR and α2C AR inhibitor respectively. We observed that Dex suppressed the increase of ADORA2B, α-SMA and collagen I mRNA levels with TGF-β stimulation, which was strongly rescued by BRL44408 but not Imiloxan HCl and JP-1302 (supplementary Fig. 3A-C). Consistently, MRC5s treated with Dex after TGF-β stimulation showed significantly decreased protein expression of ADORA2B, α-SMA and collagen I, which was blocked by BRL44408 instead of Imiloxan HCl and JP-1302 (supplementary Fig. 3D-E). In conclusion, the effect of Dex on inhibiting TGF-β-induced differentiation of lung myofibroblasts is α2A AR-dependent.

### Dex inhibits experimental pulmonary fibrosis via regulating ADORA2B

**Fig. 6:**
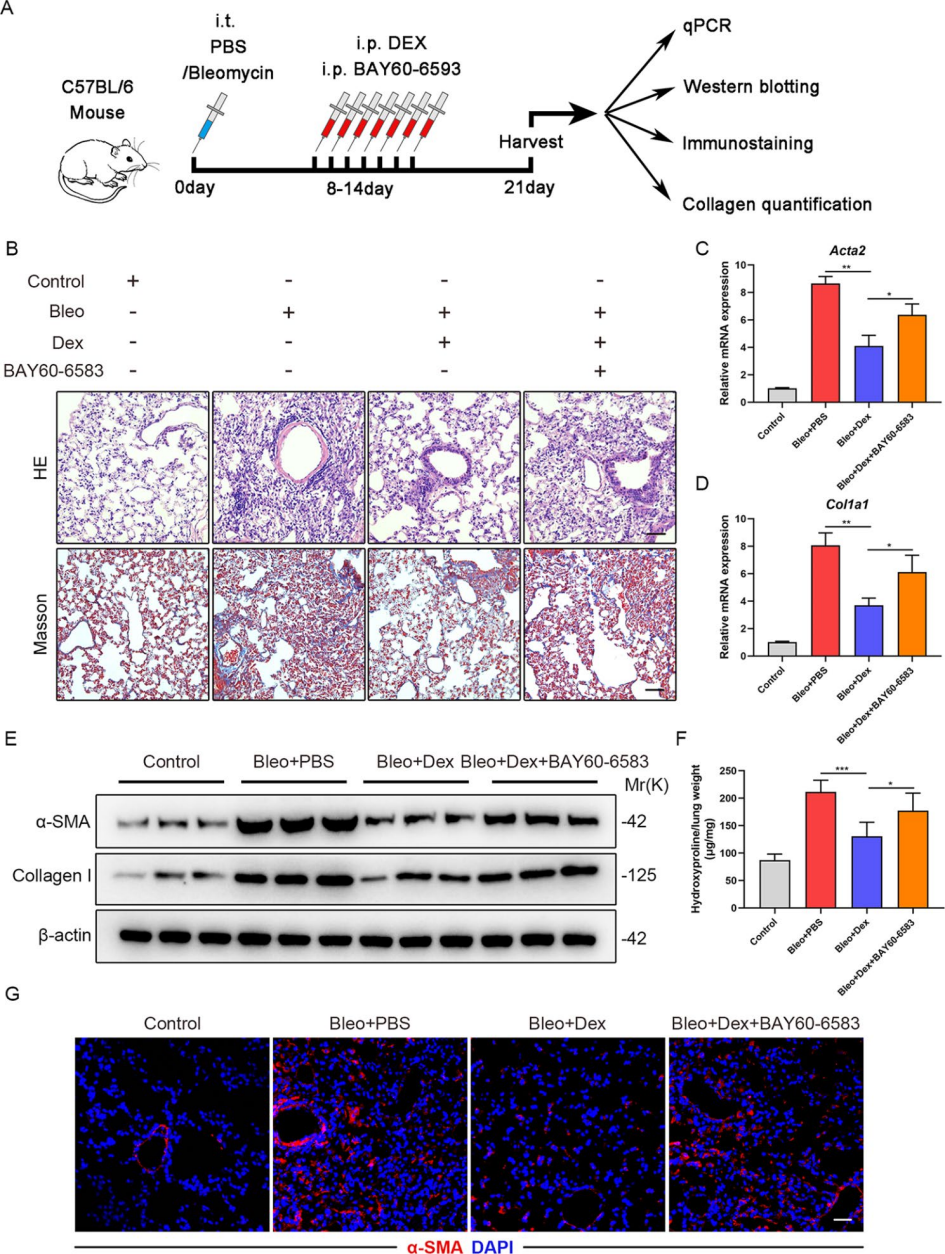
Dex inhibits experimental pulmonary fibrosis via regulating ADORA2B. (**A**) Experimental design. 8-week-old C57BL/6 mice were injected with bleomycin (3U/kg) or PBS intratracheally. Eight days later, the mice were intraperitoneal injected with Dex and BAY60-6583. Lung tissues were collected for experiments 21 days after bleomycin administration. (**B**) Representative H&E staining and Masson’s trichrome staining of lung sections from the different groups. Scale bars: 50 μm. (**C**) qPCR analysis of *Acta2* mRNA expression in the lungs of C57/BL6 mice from the different groups (n = 6 mice per group). (**D**) qPCR analysis of *Col1a1* mRNA expression in the lungs of C57/BL6 mice from the different groups (n = 6 mice per group). (**E**) Western blotting of α-SMA and Collagen I expression levels in lungs of C57/BL6 mice from the different groups. (**F**) Hydroxyproline levels in lungs of C57/BL6 mice from the different groups (n = 6 mice per group). (**G**) Representative immunofluorescence images of lung sections using anti-α-SMA (red) antibodies from the different groups. Scale bars: 50 μm. Data are presented as the mean ± SD of three independent experiments; *P < 0.05, **P < 0.01, ***P < 0.001; one-way ANOVA and Tukey’s multiple comparisons test

To further determine the molecular mechanisms by which Dex inhibits experimental pulmonary fibrosis in vivo, bleomycin-induced pulmonary fibrosis murine models were established and subjected to Dex injection intraperitoneally with or without BAY60-6583 treatment (Fig. 6A). Twenty-one days after bleomycin administration, we obtained lung tissue samples for subsequent analysis. H&E and Masson trichrome staining showed that the collagen deposition and fibrosis in lung mesenchyme from the Bleo + Dex group was slighter than those from the Bleo group, while it was apparently restored by BAY60-6583 injection (Fig. 6B). Consistently, Dex-mediated downregulation of the mRNA and protein expression of α-SMA and Collagen I in murine fibrotic lungs, was also significantly rescued by BAY60-6583 treatment (Fig. 6C-E and G). Moreover, we proved that Dex-mediated attenuation of pulmonary fibrosis was also markedly block by BAY60-6583 administration by hydroxyproline assay (Fig. 6F). Altogether, these data suggested that Dex strongly inhibits experimental pulmonary fibrosis via regulating ADORA2B in vivo.

## Discussion

Idiopathic pulmonary fibrosis (IPF) is a prevalent and progressive lung disease characterized by fibrosis, leading to irreversible deterioration of pulmonary function and potential mortality [32]. Recently, the global COVID-19 pandemic has further aggravated the burden of IPF, with an increasing incidence as a complication following severe acute respiratory syndrome coronavirus-2 (SARS-CoV-2) infection [33, 34]. Pirfenidone and nintedanib are currently the only approved treatments for IPF, but they show notable adverse effects [33, 34]. Therefore, the development of novel therapeutic strategies to decelerate or reverse the progression of IPF is in urgent and unmet need.

Dexmedetomidine (Dex), a highly selective agonist of the alpha-2 adrenoceptor (α2AR), is frequently employed as an anesthetic-sparing agent during the perioperative period [35]. Its utilization is attributed to its sedative properties, sympatholytic effects, and ability to modulate hemodynamics [35]. Accumulating studies showed that Dex exerts protective effects on lung injury and fibrosis. Daqing Ma group showed that the administration of Dex via daily intraperitoneal injections in rats resulted in a remarkable reduction in lung edema and injury because Dex protected alveolar epithelial cells from bilirubin-induced injury [35]. Jianteng Gu group found that Dex ameliorated lung cells apoptosis induced by renal ischemia-reperfusion partly via α2AR/PI3K/Akt signaling [36]. Besides, Dex treatment attenuated endotoxin-induced acute lung injury by maintaining the dynamic balance of mitochondrial fusion/fission via activating the HIF-1a/HO-1 signaling [37]. It was reported that the α2AR/Caveolin-1/p38MAPK/NF-κB axis was mainly involved in Dexmedetomidine protection against lung injury induced by intestinal ischaemia-reperfusion [38]. In our study, we surprisingly found that Dex attenuated the inflammation and fibrosis severity of lungs in pulmonary fibrosis mice. In vitro studies revealed that Dex could inhibit lung myofibroblast differentiation induced by TGF-β, which suggests that Dex may represent a potential therapeutic strategy for IPF. However, it is in great need to further investigate the effects of Dex on other cell types in lung fibrosis via sequencing and lineage tracing tools in next studies.

Mitogen-activated protein kinases (MAPK), the member of the serine-threonine kinases superfamily, is reported to be involved in a number of biologic responses and the pathogenesis of various diseases [23]. Abundant evidence indicates that the MAPK signaling plays a central role in the differentiation of myofibroblasts and the development of lung fibrosis [25, 26]. Shuichiro Maruoka colleagues found that TGF-β markedly increased JNK, p38 MAPK, and ERK phosphorylation and activity, thus leading to myofibroblastic phenotype transformation of human lung fibroblasts to myofibroblasts [25]. In addition, resveratrol inhibits bleomycin-induced pulmonary fibrosis by regulating MAPK/AP-1 pathways [39]. MAPK pathway was also activated by CuSO4 treatment, which induced the epithelial-mesenchymal transition (EMT) and pulmonary fibrosis [26]. Here, we proved that Dex significantly inhibited the activation of MAPK pathway in pulmonary fibrosis mice model and lung myofibroblasts induced by TGF-β through in vivo and in vitro experiments.

Adenosine signaling has been identified as a target for drug development since it was implicated in a number of physiological and pathological functions through activating four types of adenosine receptors, which includes A1, A2A, A2B and A3 [40]. As one of four adenosine receptors, Adenosine A2B receptor (ADORA2B) has been widely acknowledged to be involved in injury, inflammation and fibrosis of various tissues and organs [17]. Zhong and co-workers found that A2B adenosine receptor activation could promote the production of IL-6 and the differentiation of lung fibroblasts into myofibroblasts [41]. Abundant evidence has reported that ADORA2B was the upstream activator of mitogen-activated protein kinase (MAPK) pathway [17, 27]. Here, we firstly demonstrate the relationship between Dex and ADORA2B in pulmonary fibrosis and found that Dex significantly decreased the expression of ADORA2B in lung myofibroblasts in vitro and in experimental pulmonary fibrosis models. The further mechanistic studies revealed that Dex could inhibit TGF-β-mediated MAPK pathway activation and myofibroblast differentiation possibly via ADORA2B. Further exploring the molecular mechanisms of Dex modulating ADORA2B expression in pulmonary fibrosis will be of interest.

## Conclusions

**Fig. 7:**
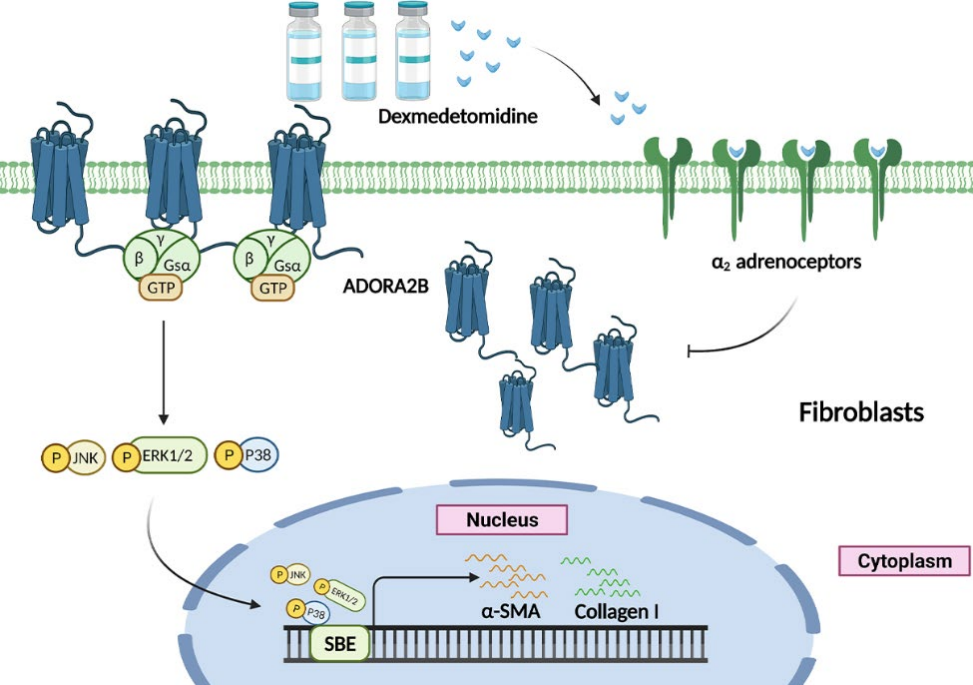
Illustration of Dexmedetomidine in the regulation of pulmonary fibrosis through the ADORA2B-mediated MAPK signaling pathway Dex inhibited TGF-β-mediated MAPK pathway activation and myofibroblast differentiation via decreasing ADORA2B expression, thus exerting a protective role in pulmonary fibrosis

Our study showed that Dex could inhibit Bleo-induced pulmonary fibrosis in mice. Dex suppressed TGF-β-mediated MAPK pathway activation and myofibroblast differentiation. Moreover, Dex inhibits myofibroblast differentiation and pulmonary fibrosis via downregulating ADORA2B expression (Fig. 7). Taken together, our findings provide important evidence for Dex as a potential therapeutic agent for pulmonary fibrosis.

### Methods

#### Animal experiments

All the animal experiments were approved by the Ethics Committee of Sun Yat-sen University (Approval number: SYSU-IACUC-2021-000837). All the C57BL/6 mice were provided by the Sun Yat-sen University Animal Center. Mice were 8–12 weeks old, fed standard food and water and housed in a standard environment at 22–24℃. Mice were randomly allocated to each group before experiments and researchers were blinded to the group arrangement. The bleomycin-induced pulmonary fibrosis model was performed as follows: mice were initially anesthetized using isoflurane and administered either bleomycin (Teva Pharmaceutical; 3 U/kg) or an equivalent volume of PBS. Dexmedetomidine was dissolved in 0.9% sterile saline and injected intraperitoneally (25 µg/kg) every day for 7 days from one week after bleomycin administration. On day 21, we obtained the lung samples for further experiments.

### Cell isolation and culture

Primary mouse lung fibroblasts were isolated following previously established protocols [42, 43]. Lungs from the C57BL/6 mice (age, 8–12 weeks) were excised into small fragments and subjected to digestion in Dulbecco’s Modified Eagle Medium with 10 mg/ml dispase (Sigma-Aldrich, D4693) and 20 mg/ml collagenase type I (Gibco, Thermo Fisher Scientific, 9001-12-1) at 37 °C for 1 h. The digested cells were sequentially filtered through 100-µm, 40-µm and 15-µm pore filters and cultured in Dulbecco’s Modified Eagle Medium containing penicillin-streptomycin and 10% fetal bovine serum (FBS, Gibco) in a 5% CO2 incubator. Fibroblasts that grew out from passages 3 to 6 were selected for subsequent experiments. TGF-β was purchased from Sigma-Aldrich.

### Histopathological evaluation

Following sacrifice, lung tissues from mice were isolated and fixed with 4% paraformaldehyde. Subsequently, the fixed tissues were subjected to a 24-hour incubation in 30% sucrose solution for saturation, enabling subsequent processing for frozen sections or paraffin embedding. The lung sections were then subjected to various staining techniques, including H&E staining, Sirius Red staining, and Masson’s trichrome staining. These staining methods were employed to evaluate and analyze the extent of inflammation and fibrosis severity within the lungs.

### Immunofluorescence staining

Following deparaffinization and antigen retrieval, or in the case of cells, lung tissues or cells were incubated overnight at 4 °C with specific antibodies. The primary and secondary antibodies utilized in the study are listed in Supplementary Table 1. To visualize the nuclei of cells, DAPI (Fluka) staining was performed. Stained tissues or cells were then imaged using a Zeiss 800 Laser Scanning Confocal Microscope or a Zeiss 880 Laser Scanning Confocal Microscope with Airyscan. ImageJ software was employed for subsequent image analysis.

### Western blotting

The protein expression levels of α-SMA, Collagen I, p-ERK, ERK, p-JNK, JNK, p-p38, p38, β-actin and ADORA2B in lung tissue samples or cultured cells were evaluated by western blotting, following established protocols [44]. The primary and secondary antibodies employed in this study are detailed in Supplementary Table 1. Quantification of all protein bands was conducted using ImageJ software, with analysis performed on data obtained from three independent blots. Full length images of immunoblots were presented in Supplementary Fig. 4.

### Real-time quantitative PCR (qPCR)

The real-time quantitative PCR were performed according to the instructions of manufacturers. Total RNA from tissues or cells was extracted using TRIzol reagent (Invitrogen), and the concentration was determined using a NanoDrop 8000 spectrophotometer. The RevertAid First Strand cDNA Synthesis Kit was purchased from Thermo Fisher Scientific and used for the cDNA synthesis. The statistical analysis was performed using FastStart Essential DNA Green Master Mix (Roche, 06924204001). All samples were analyzed in triplicate. 18 S rRNA or GAPDH was utilized as internal controls. The primer sequences employed for PCR amplification are provided in Supplementary Table 2.

### Hydroxyproline assay

To quantify the levels of hydroxyproline in frozen lung tissues, the following protocol was employed. Each frozen lung tissue sample was incubated with 250 µl of PBS and then mixed with 250 µl of 12 N HCl. The mixture was subjected to overnight incubation at 110 °C. Subsequently, the samples were neutralized using 10 N NaOH. A 100 µl aliquot from each sample was combined with 400 µl of oxidizing solution and incubated for 20 min. Following this, Ehrlich’s solution (Sigma-Aldrich) was added, and the samples were incubated at 65 °C for 30 min. The absorbance of the samples was measured at 550 nm. Standard curves were generated using trans-4-hydroxy-L-proline (Sigma-Aldrich). The levels of hydroxyproline were expressed as the amount of hydroxyproline per microgram of lung tissue.

### Statistical analysis

All data are presented as means ± standard deviations. Statistical comparisons between two groups were conducted using the unpaired Student’s t-test. For analyses involving multiple groups, one-way ANOVA with Tukey’s multiple comparison test was employed. GraphPad Software was utilized for data analysis. Statistical significance was defined as *P* < 0.05.

### Electronic supplementary material

Below is the link to the electronic supplementary material.

Supplementary Material 1

## Data Availability

Not applicable.

## References

[1] RAGHU G, WEYCKER D (2006). Incidence and prevalence of idiopathic pulmonary fibrosis[J]. Am J Respir Crit Care Med.

[2] STEELE M P, SCHWARTZ DA (2013). Molecular mechanisms in progressive idiopathic pulmonary fibrosis[J]. Annu Rev Med.

[3] WOLTERS PJ, COLLARD H R (2014). JONES K D. Pathogenesis of idiopathic pulmonary fibrosis[J]. Annu Rev Pathol.

[4] RICHELDI L, DU BOIS R M, RAGHU G (2014). Efficacy and safety of nintedanib in idiopathic pulmonary fibrosis[J]. N Engl J Med.

[5] KING T E JR, BRADFORD W Z, CASTRO-BERNARDINI S (2014). A phase 3 trial of pirfenidone in patients with idiopathic pulmonary fibrosis[J]. N Engl J Med.

[6] BELLEVILLE JP, WARD D S, BLOOR B C (1992). Effects of intravenous dexmedetomidine in humans. I. Sedation, ventilation, and metabolic rate[J]. Anesthesiology.

[7] KAMIBAYASHI T, MAZE M (2000). Clinical uses of alpha2 -adrenergic agonists[J]. Anesthesiology.

[8] SHA J, FENG X, CHEN Y (2019). Dexmedetomidine improves acute stress-induced liver injury in rats by regulating MKP-1, inhibiting NF-κB pathway and cell apoptosis[J]. J Cell Physiol.

[9] OKADA H, KURITA T, MOCHIZUKI T (2007). The cardioprotective effect of dexmedetomidine on global ischaemia in isolated rat hearts[J]. Resuscitation.

[10] JI F, LI Z (2013). Perioperative dexmedetomidine improves outcomes of cardiac surgery[J]. Circulation.

[11] YOU Z, FENG D, XU H (2012). Nuclear factor-kappa B mediates one-lung ventilation-induced acute lung injury in rabbits[J]. J Invest Surg.

[12] Chen M, Yan X T Yel et al. Dexmedetomidine Ameliorates Lung Injury Induced by Intestinal Ischemia/Reperfusion by Upregulating Cannabinoid Receptor 2, Followed by the Activation of the Phosphatidylinositol 3-Kinase/Akt Pathway. Oxid Med Cell Longev, 2020, 2020: 6120194. 10.1155/2020/6120194 PMC7327571 32655771

[13] GU J, SUN P, ZHAO H (2011). Dexmedetomidine provides renoprotection against ischemia-reperfusion injury in mice[J]. Crit Care.

[14] LI Q, CHEN C, CHEN X (2018). Dexmedetomidine attenuates renal fibrosis via α2-adrenergic receptor-dependent inhibition of cellular senescence after renal ischemia/reperfusion[J]. Life Sci.

[15] YEH Y C, WU C Y, CHENG Y J (2016). Effects of Dexmedetomidine on Intestinal Microcirculation and Intestinal Epithelial Barrier in Endotoxemic Rats[J]. Anesthesiology.

[16] AP I J, JACOBSON K A, MüLLER CE (2022). International Union of Basic and Clinical Pharmacology. CXII: Adenosine Receptors: a further Update[J]. Pharmacol Rev.

[17] VECCHIO E A, WHITE P J (2019). MAY L T. The adenosine A(2B) G protein-coupled receptor: recent advances and therapeutic implications[J]. Pharmacol Ther.

[18] VASS G, HORVáTH I (2008). Adenosine and adenosine receptors in the pathomechanism and treatment of respiratory diseases[J]. Curr Med Chem.

[19] SUN C X, ZHONG H, MOHSENIN A (2006). Role of A2B adenosine receptor signaling in adenosine-dependent pulmonary inflammation and injury[J]. J Clin Invest.

[20] LIU B, BING Q, LI S (2019). Role of A(2B) adenosine receptor-dependent adenosine signaling in multi-walled carbon nanotube-triggered lung fibrosis in mice[J]. J Nanobiotechnol.

[21] GIACOMELLI C, DANIELE S, ROMEI C (2018). The A(2B) adenosine receptor modulates the epithelial- mesenchymal transition through the balance of cAMP/PKA and MAPK/ERK pathway activation in human epithelial lung Cells[J]. Front Pharmacol.

[22] KUHN C, MCDONALD JA (1991). The roles of the myofibroblast in idiopathic pulmonary fibrosis. Ultrastructural and immunohistochemical features of sites of active extracellular matrix synthesis[J]. Am J Pathol.

[23] FANG J Y, RICHARDSON B C (2005). The MAPK signalling pathways and colorectal cancer[J]. Lancet Oncol.

[24] ARTHUR JS, LEY SC (2013). Mitogen-activated protein kinases in innate immunity[J]. Nat Rev Immunol.

[25] HASHIMOTO S, GON Y, TAKESHITA I (2001). Transforming growth Factor-beta1 induces phenotypic modulation of human lung fibroblasts to myofibroblast through a c-Jun-NH2-terminal kinase-dependent pathway[J]. Am J Respir Crit Care Med.

[26] GUO H, JIAN Z, LIU H (2021). TGF-β1-induced EMT activation via both smad-dependent and MAPK signaling pathways in Cu-induced pulmonary fibrosis[J]. Toxicol Appl Pharmacol.

[27] ANTONIOLI L, BLANDIZZI C, CSóKA B (2015). Adenosine signalling in diabetes mellitus–pathophysiology and therapeutic considerations[J]. Nat Rev Endocrinol.

[28] BREDE M, PHILIPP M, KNAUS A (2004). alpha2-adrenergic receptor subtypes - novel functions uncovered in gene-targeted mouse models[J]. Biol Cell.

[29] HUNTER JC, FONTANA D J, HEDLEY L R (1997). Assessment of the role of alpha2-adrenoceptor subtypes in the antinociceptive, sedative and hypothermic action of dexmedetomidine in transgenic mice[J]. Br J Pharmacol.

[30] MA D, HOSSAIN M, RAJAKUMARASWAMY N (2004). Dexmedetomidine produces its neuroprotective effect via the alpha 2A-adrenoceptor subtype[J]. Eur J Pharmacol.

[31] PARIS A, MANTZ J, TONNER P H (2006). The effects of dexmedetomidine on perinatal excitotoxic brain injury are mediated by the alpha2A-adrenoceptor subtype[J]. Anesth Analg.

[32] COLLARD H R, RYERSON C J, CORTE T J (2016). Acute Exacerbation of Idiopathic Pulmonary Fibrosis. An International Working Group Report[J]. Am J Respir Crit Care Med.

[33] ZHANG C, WU Z, LI JW (2021). Discharge may not be the end of treatment: pay attention to pulmonary fibrosis caused by severe COVID-19[J]. J Med Virol.

[34] GEORGE P M, WELLS A U, JENKINS RG (2020). Pulmonary fibrosis and COVID-19: the potential role for antifibrotic therapy[J]. Lancet Respir Med.

[35] CUI J, ZHAO H (2015). Dexmedetomidine attenuates Bilirubin-Induced Lung alveolar epithelial cell death in Vitro and in Vivo[J]. Crit Care Med.

[36] LI J, CHEN Q, HE X (2018). Dexmedetomidine attenuates lung apoptosis induced by renal ischemia-reperfusion injury through α(2)AR/PI3K/Akt pathway[J]. J Transl Med.

[37] SHI J, YU T, SONG K (2021). Dexmedetomidine ameliorates endotoxin-induced acute lung injury in vivo and in vitro by preserving mitochondrial dynamic equilibrium through the HIF-1a/HO-1 signaling pathway[J]. Redox Biol.

[38] XU L, LI T, CHEN Q (2021). The α2AR/Caveolin-1/p38MAPK/NF-κB axis explains dexmedetomidine protection against lung injury following intestinal ischaemia-reperfusion[J]. J Cell Mol Med.

[39] WANG J, HE F, CHEN L (2018). Resveratrol inhibits pulmonary fibrosis by regulating miR-21 through MAPK/AP-1 pathways[J]. Biomed Pharmacother.

[40] FREDHOLM B B, AP I J, JACOBSON K A (2011). International Union of Basic and Clinical Pharmacology. LXXXI. Nomenclature and classification of adenosine receptors–an update[J]. Pharmacol Rev.

[41] ZHONG H, BELARDINELLI L, MAA T (2004). A(2B) adenosine receptors increase cytokine release by bronchial smooth muscle cells[J]. Am J Respir Cell Mol Biol.

[42] WENG T, KO J, MASAMHA C P (2019). Cleavage factor 25 deregulation contributes to pulmonary fibrosis through alternative polyadenylation[J]. J Clin Invest.

[43] KöNIGSHOFF M, KRAMER M, BALSARA N (2009). WNT1-inducible signaling protein-1 mediates pulmonary fibrosis in mice and is upregulated in humans with idiopathic pulmonary fibrosis[J]. J Clin Invest.

[44] HUANG S, LAI X (2022). Asporin promotes TGF-β-induced lung myofibroblast differentiation by facilitating Rab11-Dependent recycling of TβRI[J]. Am J Respir Cell Mol Biol.

