# Supplementary material for: Dexmedetomidine alleviates pulmonary fibrosis through the ADORA2B-Mediated MAPK signaling pathway
Source: Respir Res. 2023 Aug 29;24:214. doi: 10.1186/s12931-023-02513-3 (PMC10464018; doi:10.1186/s12931-023-02513-3)
Supplement: Supplementary file 1 — Supplementary Material 1 [file 12931_2023_2513_MOESM1_ESM.docx]

**Supplementary Figures：**

**
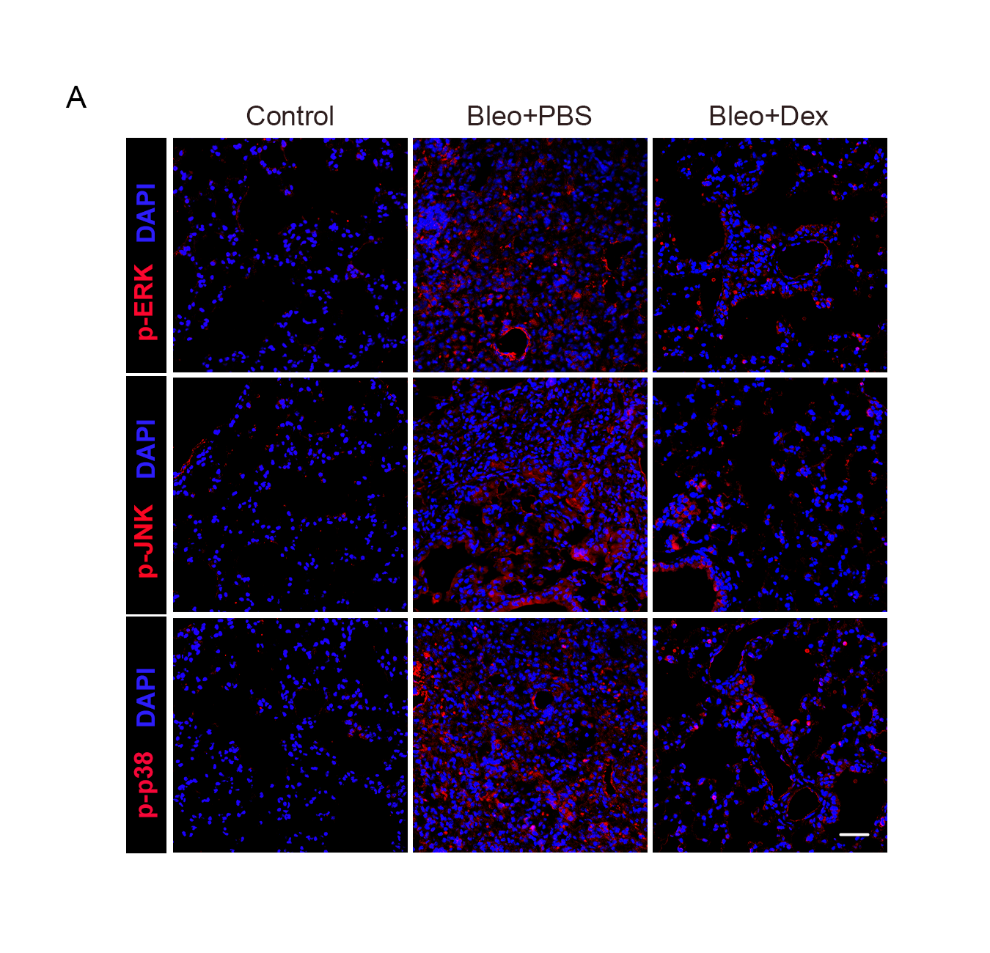
**

**Supplementary Figure 1:** **Dex inhibits** **the activation of MAPK signaling pathway in fibrotic lungs.**

Representative immunofluorescence images of lung sections using anti-p-ERK, anti-p-JNK or anti-p-p38 (red) antibodies from the different groups (n=5 mice per group; five fields assessed per sample). Scale bar: 50 µm.

**
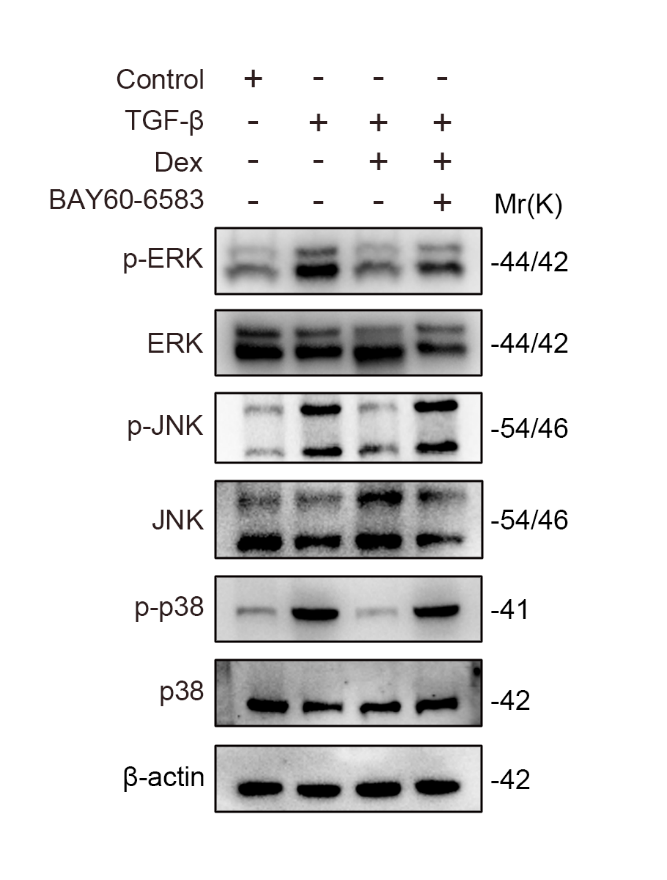
**

**Supplementary Figure 2:** **Dex** **inhibits TGF-β-mediated MAPK pathway activation and myofibroblast differentiation via regulating ADORA2B.**

Western blot analysis of p-ERK, ERK, p-JNK, JNK, p-p38 and p38 expression level in MRC5s subjected to Dex or BAY60-6583 treatment with or without TGF-β (5 ng/ml). Data are presented as the mean ± SD of three independent experiments; One-way ANOVA and Tukey’s multiple comparisons test.

**
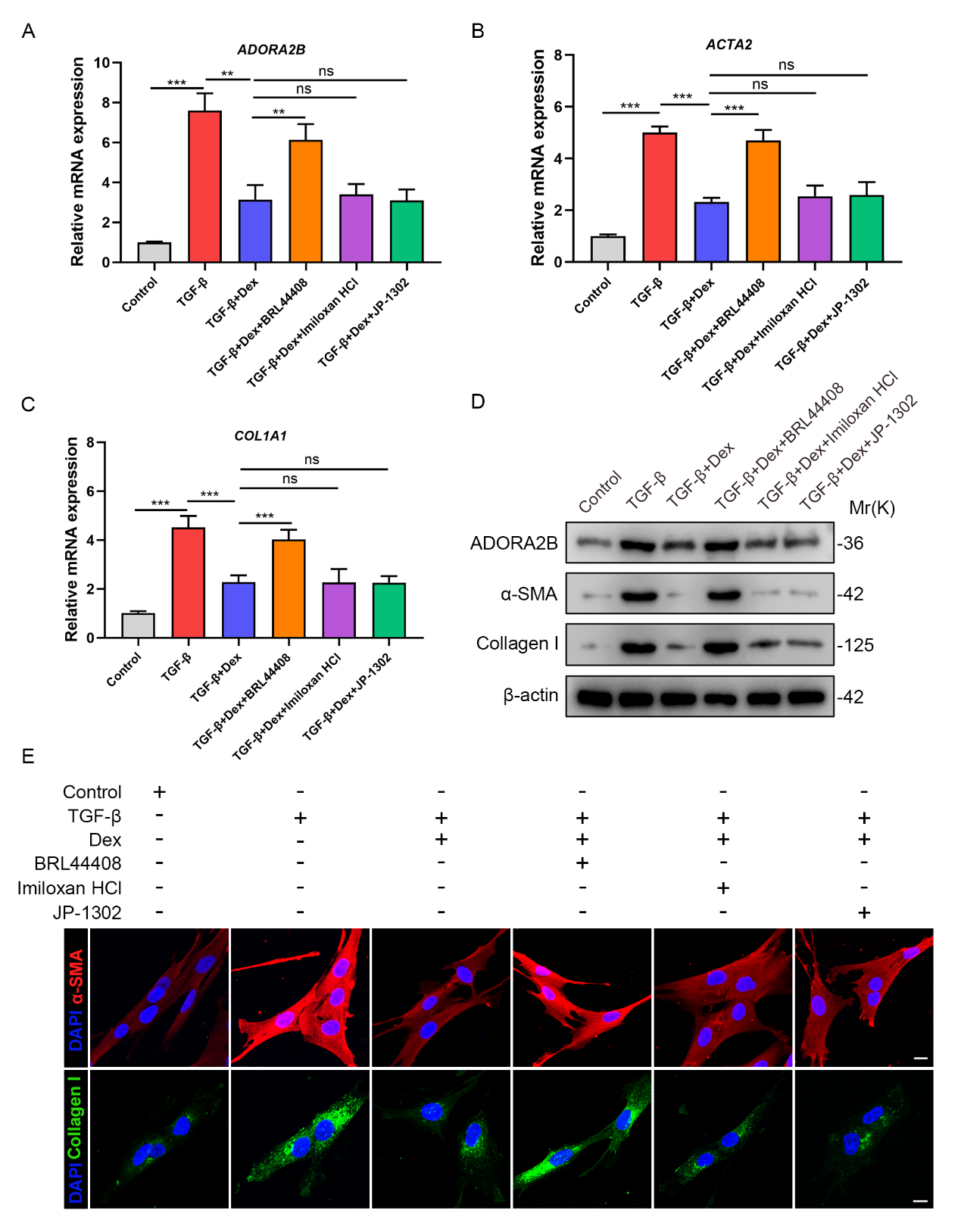
**

**Supplementary Figure 3: The role of Dex in TGF-β-induced differentiation of lung myofibroblasts is dependent on α2A adrenoreceptor**

(A) qPCR analysis of *ADORA2B* mRNA expression in MRC5s subjected to Dex, BRL44408, Imiloxan HCl or JP-1302 treatment with or without TGF-β (5 ng/ml). (B) qPCR analysis of *ACTA2* mRNA expression in MRC5s subjected to Dex, BRL44408, Imiloxan HCl or JP-1302 treatment with or without TGF-β (5 ng/ml). (C) qPCR analysis of *COL1A1* mRNA expression in MRC5s subjected to Dex, BRL44408, Imiloxan HCl or JP-1302 treatment with or without TGF-β (5 ng/ml). (D) Western blotting of ADORA2B, α-SMA and Collagen I expression levels in MRC5s subjected to Dex, BRL44408, Imiloxan HCl or JP-1302 treatment with or without TGF-β (5 ng/ml). (E) Representative immunofluorescence images of MRC5s subjected to Dex, BRL44408, Imiloxan HCl or JP-1302 treatment with or without TGF-β (5 ng/ml) using anti-α-SMA (red) or anti-Collagen I (green) antibodies. Scale bar: 20 µm. Data are presented as the mean ± SD of three independent experiments; **P<0.01, ***P<0.001, ns: no significance; one-way ANOVA and Tukey’s multiple comparisons test.

**
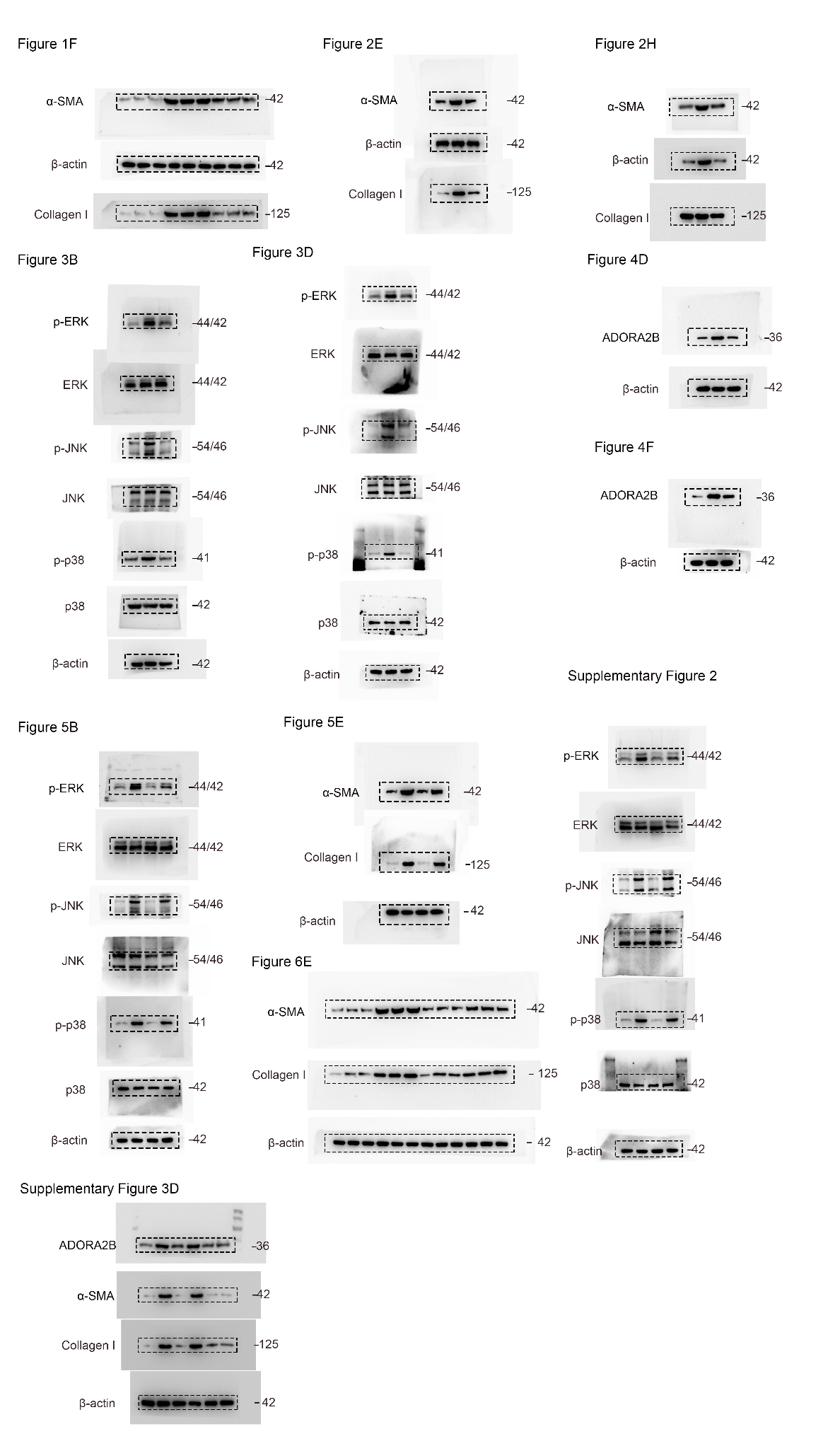
**

**Supplementary Figure 4. Full length images of immunoblots.**

**Supplementary tables:**

**Supplementary Table 1: Primary and secondary antibodies.**

| **Product** | **Catalogue Number** | **Supplier** |
| --- | --- | --- |
| **Primary antibody:** |  |  |
| **mouse Anti-β-actin** | **600081** | **Proteintech** |
| **rabbit anti-Collagen I** | **ab34710** | **Abcam** |
| **mouse anti-α-SMA** | **ab7817** | **Abcam** |
| **rabbit anti-p-ERK**  **rabbit anti-ERK**  **mouse anti-ERK**  **rabbit anti-p-JNK**  **mouse anti-p-JNK**  **rabbit anti-JNK**  **rabbit anti-p38**  **rabbit anti-p-p38**  **rabbit anti-Adenosine A2b Receptor**  **Secondary antibody:**  **anti-mouse IgG HRP-linked Ab**  **anti-rabbit IgG HRP-linked Ab**  **goat anti-mouse IgG Alexa 488**  **goat anti-rabbit IgG Alexa 488**  **goat anti-rabbit IgG Alexa 555**  **goat anti-mouse IgG Alexa 555** | **9101**  **9102**  **4696**  **4668**  **9255**  **9252**  **9212**  **4511**  **AB1589P**  **7076**  **7074**  **A11001**  **A11008**  **A21428**  **A21422** | **Cell Signaling Technology**  **Cell Signaling Technology**  **Cell Signaling Technology**  **Cell Signaling Technology**  **Cell Signaling Technology**  **Cell Signaling Technology**  **Cell Signaling Technology**  **Cell Signaling Technology**  **Merck Millipore**  **Cell Signaling Technology**  **Cell Signaling Technology**  **Invitrogen**  **Invitrogen**  **Invitrogen**  **Invitrogen** |

**Supplementary Table 2: Primer used to amplify the transcripts or genome DNA during PCR.**

| **Gene** | **Sequences (5′ to 3′)** | **application** |
| --- | --- | --- |
| **hADORA2B**  **mAdora2b** | **Forward: 5’-GGGGTGGAACAGTAAAGACAG-3’**  **Reverse: 5’-CAGCAGCTTTCATTCGTGGTT-3’**  **Forward: 5’-GGGGTGGAACAGTAAAGACAG-3’**  **Reverse:5’-CAGCAGCTTTCATTCGTGGTT-3’** | **qPCR**  **qPCR** |
| **hADORA2A**  **mAdora2a** | **Forward: 5’-CATGCTAGGTTGGAACAACTGC-3’**  **Reverse: 5’-AGATCCGCAAATAGACACCCA-3’**  **Forward: 5’-TTCCACTCCGGTACAATGGC-3’**  **Reverse:5’-CGATGGCGAATGACAGCAC-3’** | **qPCR**  **qPCR** |
| **hADORA1**  **mAdora1**  **hADORA3**  **mAdora3**  **mActa2**  **hACTA2** | **Forward: 5’-CCACAGACCTACTTCCACACC-3’**  **Reverse: 5’-TACCGGAGAGGGATCTTGACC-3’**  **Forward: 5’-TTCATCGTATCCCTGGCGGTA-3’**  **Reverse:5’-TCTGTGGCCCAATGTTGATAAG-3’**  **Forward: 5’-GAGTGGAGCACGTAAGCACT-3’**  **Reverse:5’-ATGGAGTTGGCATGGGACAG-3’**  **Forward: 5’-CCGACAACACCACGGAGACG-3’**  **Reverse:5’-AGCTTGACCACCCAGATGACC-3’**  **Forward: 5’-TGAGACCTTCAATGTCCCCGC-3’**  **Reverse: 5’-TCACACCATCTCCAGAGTCCAGC-3’**  **Forward: 5’-AAAAGACAGCTACGTGGGTGA-3’**  **Reverse: 5’-GCCATGTTCTATCGGGTACTTC-3’** | **qPCR**  **qPCR**  **qPCR**  **qPCR**  **qPCR**  **qPCR** |
| **mcol1a2**  **mcol1a1**  **mFn** | **Forward: 5’-CCCAGAGTGGAACAGCGATT-3’**  **Reverse: 5’-ATGAGTTCTTCGCTGGGGTG -3’**  **Forward:** **5’-TGACTGGAAGAGCGGAGAGT-3’**  **Reverse:** **5’-GTTCGGGCTGATGTACCAGT-3’**  **Forward:** **5’-GGTGTAGCACAACTTCCAATTACG-3’**  **Reverse:** **5’-GGAATTTCCGCCTCGAGTCT-3’** | **qPCR**  **qPCR**  **qPCR** |
| **m18s**  **hGAPDH** | **Forward: 5’-GTAACCCGTTGAACCCCATT-3’**  **Reverse: 5’-CCATCCAATCGGTAGTAGCG-3’**  **Forward: 5’-AGGTCGGTGTGAACGGATTTG-3’**  **Reverse: 5’-TGTAGACCATGTAGTTGAGGTCA-3’** | **qPCR**  **qPCR** |
